# Association between Self-Perceived Social Support in the Workplace and the Presence of Depressive/Anxiety Symptoms

**DOI:** 10.3390/ijerph181910330

**Published:** 2021-09-30

**Authors:** Juyeon Oh, Seunghyun Lee, Juho Sim, Seunghan Kim, Ara Cho, Byungyoon Yun, Jin-Ha Yoon

**Affiliations:** 1 Department of Information Statistics, Wonju Christian Yonsei University, Wonju 26493, Korea; wbdys33@yonsei.ac.kr; 2 The Institute for Occupational Health, Yonsei University College of Medicine, Seoul 03722, Korea; jay1250@yuhs.ac (S.L.); flyinyou@yuhs.ac (J.-H.Y.); 3 Department of Public Health, Graduate School, Yonsei University, Seoul 03722, Korea; yodasim@yuhs.ac; 4 Department of Preventive Medicine, Yonsei University College of Medicine, Seoul 03722, Korea; hanpurple12@gmail.com; 5 Department of Occupational Health, Graduate School of Public Health, Yonsei University, Seoul 03722, Korea; aracho88@yuhs.ac

**Keywords:** social support, depression, anxiety, wage workers

## Abstract

This study aimed to investigate the association of support from colleagues and supervisors at the workplace on depressive and anxiety symptoms in wage earners from Korea. The data used in this study were from the fifth Korean Working Conditions Survey (KWCS) conducted in 2017 and analyzed using a multivariate logistic regression model. Furthermore, we measured the odds ratios (ORs) and 95% confidence intervals (CIs) of depressive and anxiety symptoms by stratifying covariates. The ORs of depressive and anxiety symptoms for the “non-support” group were higher than for the “support group” in terms of support from both colleagues and supervisors. The results of the stratified analysis of covariates, male, young, highly-educated, full-time, and white-collar groups were associated with the lack of support. Support from colleagues and supervisors was significantly associated with the Korean wage worker’s mental health—depressive and anxiety symptoms, respectively. Further longitudinal and clinical studies on the relationship between mental health and support at the workplace are required.

## 1. Introduction

Mental health is generally considered an important aspect of public health. Depression and anxiety are common mental health problems that can lead to chronic diseases such as obesity [1], diabetes [2], and cardiovascular diseases [3], and have a significant impact on stress [4], which can subsequently cause various other diseases [5]. Furthermore, the treatment of mental health problems, including depression and anxiety, is expensive. A study estimated the overall cost of major depressive disorders in the United States to be $236.6 billion in 2010 and $326.2 billion in 2018 [6]. The average overall medical cost of treatment of a person diagnosed with anxiety disorders was $6475, with generalized anxiety disorder (GAD) patients paying an extra $2138; patients with co-existing depression or other anxiety disorders paid an additional $1900 [7]. Moreover, depressive disorder is a major public health problem in Korea where the overall cost was estimated to be $4049 million with $152.6 million direct cost in 2005 [8].

In the workplace, the mental health of workers is an important issue, as it reduces work productivity [9] and affects workers’ stress levels, and stressed workers are more likely to experience work disruptions and industrial disasters [10]. The workplace proportion of economic costs due to workers’ major depressive disorder in the United States increased from 48% (2010) to 61% (2018) [6]. Additionally, Zomer et al. conducted a study of productivity loss due to depression in Korea, and the result showed that over 55,000 discounted years of life, which is equal to $122 billion in GDP loss, were lost because of depression [11].

To improve the mental health of workers, international and regional organizations have formulated several methods of prevention and management [12]. The World Health Organization (WHO) announced the Global Framework for Healthy Workplaces with the goal of offering guidance for the protection and promotion of workers’ health, safety, and well-being, as well as the workplace’s sustainability. Furthermore, several regional organizations have been established to facilitate cooperation, political and economic integration, and conversations among governments in a specific geographic area. Some have implemented policies aimed at promoting mental health and well-being and preventing stress and associated diseases at the regional level, with the goal of merging or coordinating national efforts [12].

Despite these efforts, however, mental diseases in the workplace remain a significant problem that requires intervention and prevention. Factors affecting workers’ mental health include long working hours, shift work, income levels, hazardous working conditions, labor intensity, and organizational support [12,13]. Previous studies have reported that organizational support positively affects work performance, occupational productivity [9], and the mental health, including depression and anxiety, of workers [14,15,16,17]. Given that employees spend the majority of their time in the workplace, support from colleagues and supervisors has a significant impact on workers’ mental fatigue, stress, and job satisfaction [18,19].

However, most studies have focused on specific occupations and single outcomes [14,15,16]. This study, thus, aims to examine the relationship between self-perceived support from supervisors or colleagues and self-perceived general emotional distress states, including depressive and anxiety symptoms, using the Korean Working Conditions Survey (KWCS), which is a nationally representative sample.

## 2. Methods

### 2.1. Data

This study was based on data acquired from the fifth KWCS, conducted by the Occupational Safety & Health Research Institute (OSHRI), to better understand the types of employment, status of employment, occupational hazards, and working environment of participants. The study sample included employed individuals selected from across the country using multistage systematic cluster sampling methods. The survey was conducted via computer-assisted face-to-face interviews during house visits by trained interviewers. A total of 50,205 individuals participated in the fifth KWCS; we investigated 19,849 participants after excluding those who were self-employed, who were over 55 years of age, which is a low mandatory retirement age of Korea [20], or who supplied incomplete information.

### 2.2. Main Variables

To identify the self-perceived general emotional distress state of participants, the questions related to the presence of depressive/anxiety symptoms were used. We used the questions “In the past 12 months, have you had any health problems such as depression?” and “In the past 12 months, have you had any health problems such as anxiety?”. The participants who responded “Yes” to the questions were classified as having depressive/anxiety symptoms, respectively. To identify the level of support from colleagues and bosses at the workplace, we used the questions “Do your colleagues help and support you?” and “Does your supervisor help and support you?”. Participants answered these questions on a 5-point Likert scale (all the time (4), almost all of the time (3), sometimes (2), almost never (1), and never (0)). These responses were divided into two categories: support group and non-support group. Workers who responded “all of the time”, “almost all of the time”, and “sometimes” were categorized as the “support group”, whereas workers who responded “almost never” and “never” were categorized as the “non-support group”.

### 2.3. Covariates

Potential confounders and covariates included sociodemographic factors, such as age and sex, socioeconomic factors including monthly income (grouped as quartiles) and highest level of education: below elementary, middle school, high school, and over university (the first three categorized as “Low”, and the fourth categorized as “High”). Factors pertaining to working environment included employment status (full-time included regular workers, part-time included temporary workers and day laborers), work duration (under and over 5 years), working hours (a week; below 40 h, 41–52 h, and over 53 h), and shift work (using the question “Do you work shifts?”). Occupational category was classified as three groups: white-collar (managers, professionals, and office workers), pink-collar (service workers and sales workers), and blue-collar (skilled workers, machine operators, and assembly workers).

### 2.4. Statistical Analysis

Statistical analysis was performed using R version 4.1.0 (R Foundation for Statistical Computing, Vienna, Austria). Chi-square tests for categorical variables and T-tests for continuous variables were used to compare differences between baseline characteristics of the study population by colleagues’ support and supervisor’s support. The odds ratios (ORs) and 95% confidence intervals (95% CIs) for depressive symptoms and anxiety by support from colleagues and the supervisor were estimated using a multivariate logistic regression model. The ORs were adjusted using socioeconomic factors including age, sex, income, and education in Model1. In Model2, ORs were adjusted using factors pertaining to the work environment including employment status, work duration, working hours, shift work, and occupational category with variables of Model1. Furthermore, subgroup analyses stratified by age, sex, education, employment status, and occupational category were performed as a sensitivity analysis. A forest plot of each outcome was drawn with ORs and 95% CIs.

## 3. Results

Baseline characteristics of workers are summarized in Table 1. In our study, we found that 13,518 of 19,849 participants answered they were supported by their colleagues, and 12,603 participants reported receiving support from their supervisor. Among the total number of workers, 49.0% were males, and the mean and standard deviation of age were 39.63 and 9.51, respectively. The depressive and anxiety symptom prevalence of all participants was 2.01% and 2.62%, respectively. Among sex, more males than females responded that they were supported by colleagues (70.49% males and 65.81% females). Participants with higher levels of income and education reported receiving support from their colleagues. More full-time workers (69.63%) received support from colleagues compared to part-time workers (59.23%). People with more than five years of work experience received more support from their colleagues, and those working under 40 h a week showed a higher degree of support. depressive and anxiety symptoms were also significantly associated with support from colleagues (*p* < 0.001). The outcomes of support from supervisors were similar to those of colleagues, except that the relationship between age and support from the supervisor was not significant.

Table 2 summarizes the estimated ORs and 95% CIs of the final model using the multivariate logistic regression model, for depressive and anxiety symptoms. Referring to colleagues’ support, the non-support group was at significantly higher ORs of depressive and anxiety symptoms compared with the support group (1.61 (1.31–1.97), 1.69 (1.41–2.02), respectively). Regarding supervisors’ support, compared to the support group, ORs of depressive and anxiety symptoms for the non-support group were 1.71 (1.40–2.09) and 1.91 (1.60–2.27), respectively, and statistically significant. Detailed contents of all logistic regression models are shown in Supplementary Tables S1–S4.

**Figure 1:**
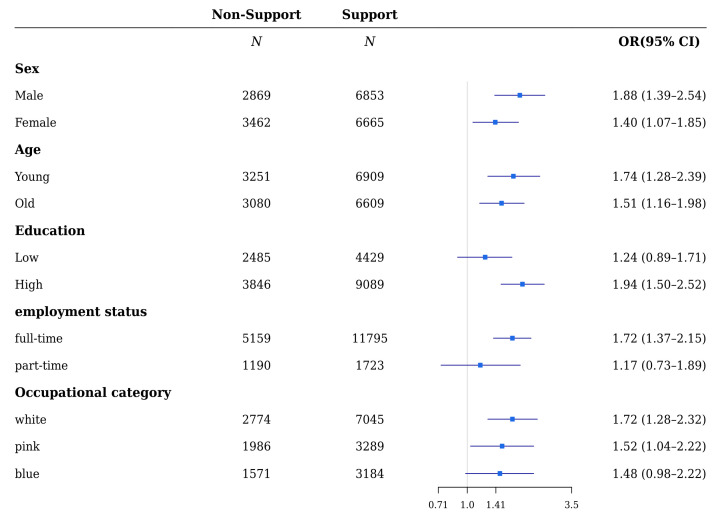
Subgroup analyses of the risk of depressive symptoms by support from colleagues in wage workers.

**Figure 2:**
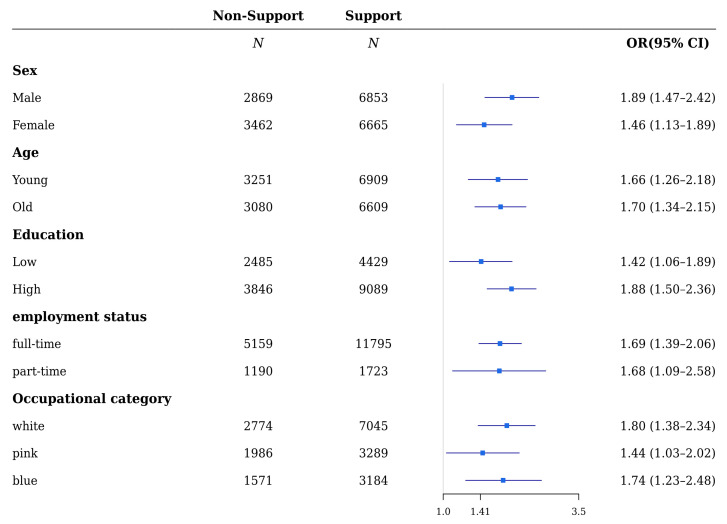
Subgroup analyses of the risk of anxiety symptoms by support from colleagues in wage workers.

Based on the analysis of each variable’s stratification, regarding colleagues’ support (Figure 1 and Figure 2), the ORs of depressive and anxiety symptoms by lack of support were significant in both males and females (ORs were 1.88 (1.39–2.54), 1.40 (1.07–1.85) and 1.89 (1.47–2.42), 1.46 (1.13–1.89), respectively). The ORs of depressive and anxiety symptoms in both the young and old who were in the non-support group were statistically significant (1.74 (1.28–2.39), 1.51 (1.16–1.98) and 1.66 (1.26–2.18), 1.70 (1.34–2.15), respectively). The relationship between depressive or anxiety symptoms and lack of support was statistically significant, except for depressive symptoms in the group of people with low education levels. The ORs of depressive and anxiety symptoms for people with low and high education levels were 1.24 (0.89–1.71), 1.94 (1.50–2.52) and 1.42 (1.06–1.89), 1.88 (1.50–2.36), respectively. The association of lack of support with depressive and anxiety symptoms was significant in both full-time and part-time workers, except for depressive symptoms in part-time workers: ORs of depressive and anxiety symptoms were 1.72 (1.37–2.15), 1.17 (0.73–1.89) and 1.69 (1.39–2.06), 1.68 (1.09–2.58) for full-time and part-time workers, respectively. Furthermore, lack of support was significant for depressive and anxiety symptoms in both white-, pink-, and blue-collared workers, except for depressive symptoms in blue-collared workers (ORs of depressive and anxiety symptoms were 1.72 (1.28–2.32), 1.52 (1.04–2.22), 1.48 (0.98–2.22), 1.80 (1.38–2.34), and 1.44 (1.03–2.02), 1.74 (1.23–2.48) for white-, pink-, and blue-collared workers, respectively).

**Figure 3:**
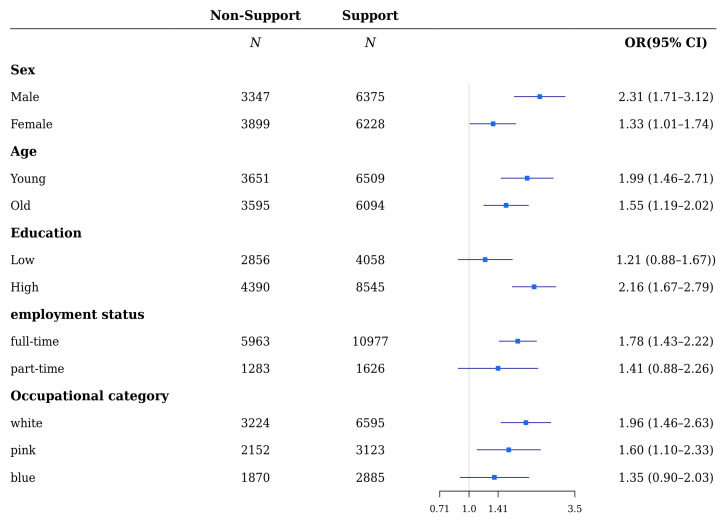
Subgroup analyses of the risk of depressive symptoms by support from supervisors of wage workers.

**Figure 4:**
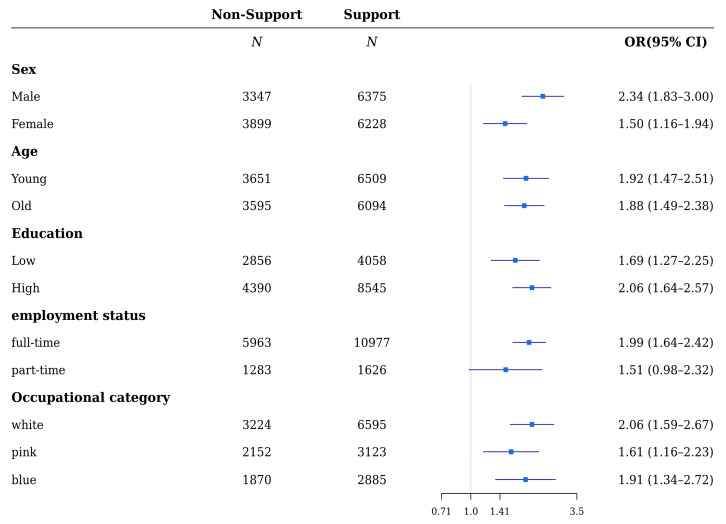
Subgroup analyses of the risk of anxiety symptoms by support from supervisors of wage workers.

Referring to the supervisor’s support (Figure 3 and Figure 4), the relationship between the non-support group and mental health including depressive and anxiety symptoms was significant in both males and females (ORs were 2.31 (1.71–3.12), 1.33 (1.01–1.74) and 2.34 (1.83–3.00), 1.50 (1.16–1.94) for males and females with depressive and anxiety symptoms, respectively). In both the young and old, lack of support was significant for depressive and anxiety symptoms (ORs were 1.99 (1.46–2.71), 1.55 (1.19–2.02) and 1.92 (1.47–2.51), 1.88 (1.49–2.38) for the young and old with depressive and anxiety symptoms, respectively). The relationship between lack of support and depressive and anxiety symptoms was significant in both groups with high and low education levels, except for depressive symptoms in the group with low-education (ORs of depressive and anxiety symptoms were 1.21 (0.88–1.67), 2.16 (1.67–2.79) and 1.69 (1.27–2.25), 2.06 (1.64–2.57) for those with low and high levels of education, respectively). Except for the part-time workers’ group, the ORs of depressive and anxiety symptoms for full-time workers who were in the non-support group were statistically significant (ORs for depressive and anxiety symptoms were 1.78 (1.43–2.22), 1.41 (0.88–2.26) and 1.99 (1.64–2.42), 1.51 (0.98–2.32) for full-time and part-time workers, respectively). Moreover, the association of depressive and anxiety symptoms and lack of support was statistically significant, except for depressive symptoms in the blue-collared workers’ group (ORs of depressive and anxiety symptoms were 1.96 (1.46–2.63), 1.60 (1.10–2.33), 1.35 (0.90–2.03), 2.03 (1.59–2.67), and 1.61 (1.16–2.23), 1.91 (1.34–2.72) for white-, pink-, and blue-collared workers, respectively).

## 4. Discussion

In this study, we investigated whether support from colleagues and supervisors affected the prevalence of depressive and anxiety symptoms in wage-earners. Workers bereft of support from colleagues or supervisors had significantly higher ORs of depressive and anxiety symptoms, a finding consistent with those of previous studies. Income levels—low-middle and high-middle—that showed low ORs of depressive symptoms were significantly related to support from colleagues and supervisors. In addition, support had a significant association between working hours and anxiety symptoms. Moreover, supervisors’ support was more highly associated with mental health than colleagues’ support was.

Stratified analyses were performed with the variables including sex, age, education, employment status, and occupational category. Support at the workplace was more significant for male, young, highly educated, full-time, and white-collared workers. In general, female workers were more likely than male workers to experience depressive and anxiety symptoms due to occupational factors [21,22,23]. However, we found that male workers had higher ORs of psychological support in the workplace for depressive and anxiety symptoms than female workers. Indeed, a study showed male workers were more effective than female workers in providing emotional support for depressive symptoms [24]. Male workers might tend to underestimate or be oblivious to their mental health problems [25,26]. Therefore, it is implied that psychological support in the workplace is important for male as well as female workers.

As for age, the younger workers were more affected by support than the older workers; a study [27] suggested that when offered support, the young had better mental health resilience and utilization of mental health care services than the older people [28]. Robust support in the workplace for middle-aged workers with low psychological resilience and utilization of mental health care services may be required.

When it comes to the occupational category, a study suggested that white-collar work was considered meaningful, including inducing in workers a sense of unity with and service to others, and thus more important than pink- or blue-collar work [29]. Since white-collar workers value person–organization fit and commitment to the organization [30], support from its members is more important for them than for blue- and pink-collar workers; this observation is reflected in the OR values in our study findings.

Lack of support has a negative relationship to the workplace environment, in addition to impacting workers’ mental health [31,32,33,34]. Repeated exposure to negative occupational condition or environments, including lack of support from colleagues and supervisors, increases job-related stress, depression, and anxiety; this could lead to making workers less attentive at work, thereby resulting in unsafe behavior [35]. Heinrich’s domino theory emphasizes that reducing accident frequency rate, which could be caused by psychological factors and unsafe behavior, would achieve an equivalent reduction in injury severity [36]. Therefore, managing a worker’s mental health and preventing resultant accidents is a crucial strategy for reducing risk of serious industrial disasters.

Enormous efforts are being made to prevent mental health problems for workers worldwide. Several nations have reported that occupational health services (OHS) for workers’ mental health have proven to have positive effects [33]. According to the International Labor Organization, the success of an organization is based on its environment: workers in a safe and supportive workplace are healthier, which contributes to lower absenteeism, increased motivation, and increased productivity [37]. Workplace improvements contribute to national improvement and are a significant part of effective economic and social strategies [38]. Hence, as support at the workplace can reduce and prevent depression and anxiety in workers, measures such as serious attention, continuous management, and national improvement toward the same are required.

This study has a few limitations. First, it was based on a cross-sectional analysis; we could not infer the causality of support and mental health because of unexpected intermediary factors that may have associations with outcomes. We could not exclude the possibility of workers being afflicted with depressive and anxiety symptoms before joining work, which might have caused a negative assessment of support. Second, since the data in this study are based on self-reports, they might have a possibility of recall bias. Furthermore, mental symptoms and social support were measured by a single question each, which could be biased due to insufficient understanding of the question. However, KWCS, which was used in our study, is based on the European Working Conditions Surveys and showed high external and content validity and reliability [39].

Despite these limitations, this study has several strengths. First, the data that we used were from KWCS, a representative national survey that analyzes working environment and worker health problems and offers reliable samples of Korean workers. Second, most previous studies have focused either on depression or anxiety or specific occupations. Moreover, a prior study focused on only one kind of support, either from colleagues or bosses, as a factor influencing and affecting mental health. In this study, we investigated support from coworkers as well as supervisors being associated with significantly decreased depressive and anxiety symptoms, respectively.

## 5. Conclusions

This study demonstrated the relationship of depressive symptoms as well as anxiety symptoms with workers who receive support at the workplace. Protection of workers’ health has become a global issue. Given that mental disorders can be prevented at the workplace, global attention and continuous management are required to improve workers’ mental health. Further longitudinal and clinical studies are necessary to overcome the limitations of the present study.

## Figures and Tables

**Table 1:** Baseline characteristics of workers by support from colleagues or supervisors.

Variable	Support from Colleagues		*p*	Support from Supervisor		*p*
	No	Yes		No	Yes	
**Age**			0.012			0.235
Mean (SD)	39.37 (9.85)	39.74 (9.35)		39.73 (9.76)	39.56 (9.37)	
**Sex**			<0.001			<0.001
Male	2869 (29.51%)	6853 (70.49%)		3347 (34.43%)	6375 (65.57%)	
Female	3462 (34.19%)	6665 (65.81%)		3899 (38.50%)	6228 (61.50%)	
**Income**			<0.001			<0.001
Low	1647 (40.77%)	2393 (59.23%)		1796 (44.46%)	2244 (55.54%)	
Low-Middle	1766 (35.51%)	3207 (64.49%)		1976 (39.73%)	2997 (60.27%)	
High-Middle	1732 (29.53%)	4134 (70.47%)		2039 (34.76%)	3827 (65.24%)	
High	1186 (23.86%)	3784 (76.14%)		1435 (28.87%)	3535 (71.13%)	
**Education**			<0.001			<0.001
Low	2485 (35.94%)	4429 (64.06%)		2856 (41.31%)	4058 (58.69%)	
High	3846 (29.73%)	9089 (70.27%)		4390 (33.94%)	8545 (66.06%)	
**Employment status**			<0.001			<0.001
full-time	5159 (30.30%)	11,795 (69.63%)		5963 (35.20%)	10,977 (64.80%)	
part-time	1190 (40.78%)	1723 (59.23%)		1283 (44.10%)	1626 (55.90%)	
**Work duration**			<0.001			<0.001
<5 year	4208 (34.44%)	8009 (65.56%)		4721 (38.64%)	7496 (61.36%)	
≥5 year	2123 (27.82%)	5509 (72.18%)		2525 (33.08%)	5107 (66.92%)	
**Working Hour**			<0.001			<0.001
≤40 h	3547 (30.88%)	7939 (69.12%)		4061 (35.36%)	7425 (64.64%)	
41–52 h	1958 (33.72%)	3848 (66.28%)		2215 (38.15%)	3591 (61.85%)	
≥53 h	826 (32.30%)	1731 (67.70%)		970 (37.94%)	1587 (62.06%)	
**Shift Work**			0.209			0.442
No	5629 (32.05%)	11,935 (67.95%)		6429 (36.60%)	11,135 (63.40%)	
Yes	702 (30.72%)	1583 (69.28%)		817 (35.75%)	1468 (64.25%)	
**Occupational category**			<0.001			<0.001
white-collar	2774 (28.25%)	7045 (71.75%)		3224 (32.83%)	6595 (67.17%)	
pink-collar	1986 (37.65%)	3289 (62.35%)		2152 (40.80%)	3123 (59.20%)	
blue-collar	1571 (33.04%)	3184 (66.96%)		1870 (39.33%)	2885 (60.67%)	
**Depression**			<0.001			<0.001
No	6158 (31.66%)	13,292 (68.34%)		7046 (36.23%)	12,404 (63.77%)	
Yes	173 (43.36%)	226 (56.64%)		200 (50.13%)	199 (49.87%)	
**Anxiety**			<0.001			<0.001
No	6105 (31.59%)	13,223 (68.41%)		6977 (36.10%)	12,351 (63.90%)	
Yes	226 (43.38%)	295 (56.62%)		269 (51.63%)	252 (48.37%)	

Abbreviation: OR; odds ratio, CI; confidence interval.

**Table 2:** Estimated ORs and 95% CIs for depression and anxiety with the support of colleagues and supervisor.

Variable	Support from Colleagues		Support from Supervisor	
	Depression	Anxiety	Depression	Anxiety
	OR (95% CI)		OR (95% CI)	
(Intercept)	0.01 (0.00–0.02)	0.01 (0.01–0.02)	0.01 (0.00–0.02)	0.01 (0.01–0.02)
**Support**				
Yes	1.00 (reference)	1.00 (reference)	1.00 (reference)	1.00 (reference)
No	1.61 (1.31–1.97)	1.69 (1.41–2.02)	1.71 (1.40–2.09)	1.91 (1.60–2.27)
**Age**	1.02 (1.01–1.03)	1.02 (1.01–1.03)	1.02 (1.01–1.03)	1.01 (1.00–1.03)
**Sex**				
Male	1.00 (reference)	1.00 (reference)	1.00 (reference)	1.00 (reference)
Female	1.01 (0.79–1.29)	0.89 (0.71–1.10)	1.01 (0.79–1.29)	0.89 (0.72–1.11)
**Income**				
Low	1.00 (reference)	1.00 (reference)	1.00 (reference)	1.00 (reference)
Low-Middle	0.72 (0.53–0.97)	0.78 (0.59–1.04)	0.72 (0.54–0.98)	0.79 (0.59–1.04)
High-Middle	0.66 (0.47–0.91)	0.77 (0.57–1.04)	0.66 (0.47–0.91)	0.77 (0.57–1.04)
High	0.73 (0.50–1.07)	1.16 (0.83–1.62)	0.74 (0.50–1.08)	1.18 (0.84–1.65)
**Education**				
Low	1.00 (reference)	1.00 (reference)	1.00 (reference)	1.00 (reference)
High	0.99 (0.77–1.28)	0.99 (0.79–1.24)	1.00 (0.77–1.29)	0.99 (0.79–1.25)
**Employment status**				
full-time	1.00 (reference)	1.00 (reference)	1.00 (reference)	1.00 (reference)
part-time	1.15 (0.86–1.53)	1.23 (0.94–1.59)	1.16 (0.87–1.55)	1.23 (0.95–1.60)
**Work duration**				
<5 year	1.00 (reference)	1.00 (reference)	1.00 (reference)	1.00 (reference)
≥5 year	1.03 (0.80–1.31)	1.09 (0.87–1.35)	1.03 (0.80–1.31)	1.09 (0.88–1.35)
**Working hour**				
≤40 h	1.00 (reference)	1.00 (reference)	1.00 (reference)	1.00 (reference)
41–52 h	1.17 (0.93–1.47)	1.47 (1.21–1.79)	1.17 (0.93–1.47)	1.47 (1.20–1.78)
≥53 h	1.35 (0.99–1.84)	1.32 (1.00–1.74)	1.34 (0.98–1.83)	1.30 (0.99–1.72)
**Shift work**				
No	1.00 (reference)	1.00 (reference)	1.00 (reference)	1.00 (reference)
Yes	1.01 (0.73–1.39)	1.21 (0.93–1.58)	1.01 (0.73–1.38)	1.23 (0.95–1.60)
**Occupational category**				
white-collar	1.00 (reference)	1.00 (reference)	1.00 (reference)	1.00 (reference)
pink-collar	0.90 (0.68–1.19)	1.04 (0.81–1.33)	0.91 (0.69–1.20)	1.05 (0.82–1.34)
blue-collar	0.90 (0.66–1.22)	0.95 (0.73–1.24)	0.90 (0.66–1.22)	0.95 (0.73–1.23)

## Data Availability

Data are available in a publicly accessible repository that does not issue DOIs. These data can be found here: https://oshri.kosha.or.kr/oshri/researchField/downWorkingEnvironmentSurvey.do.

## Supplementary Materials

The following are available online at https://www.mdpi.com/article/10.3390/ijerph181910330/s1, Table S1: Multiple logistic regression model of depressive styptom and colleagues support, Table S2: Multiple logistic regression model of anxiety styptom and colleagues support, Table S3: Multiple logistic regression model of depressive styptom and supervisor support, Table S4: Multiple logistic regression model of anxiety styptom and supervisor support.

## References

[1] Milaneschi Y., Simmons W.K., van Rossum E.F.C., Penninx B.W. (2019). Depression and obesity: Evidence of shared biological mechanisms. Mol. Psychiatry.

[2] Yu M., Zhang X., Lu F., Fang L. (2015). Depression and risk for diabetes: A meta-analysis. Can. J. Diabetes.

[3] Zhang Y., Chen Y., Ma L. (2018). Depression and cardiovascular disease in elderly: Current understanding. J. Clin. Neurosci..

[4] Fattouh N., Hallit S., Salameh P., Choueiry G., Kazour F., Hallit R. (2019). Prevalence and factors affecting the level of depression, anxiety, and stress in hospitalized patients with a chronic disease. Perspect. Psychiatr. Care.

[5] Yaribeygi H., Panahi Y., Sahraei H., Johnston T.P., Sahebkar A. (2017). The impact of stress on body function: A review. EXCLI J..

[6] Greenberg P.E., Fournier A.A., Sisitsky T., Simes M., Berman R., Koenigsberg S.H., Kessler R.C. (2021). The economic burden of adults with major depressive disorder in the United States (2010 and 2018). Pharmacoeconomics.

[7] Marciniak M.D., Lage M.J., Dunayevich E., Russell J.M., Bowman L., Landbloom R.P., Levine L.R. (2005). The cost of treating anxiety: The medical and demographic correlates that impact total medical costs. Depress. Anxiety.

[8] Chang S.M., Hong J.P., Cho M.J. (2012). Economic burden of depression in south korea. Soc. Psychiatry Psychiatr. Epidemiol..

[9] Park K.O., Wilson M.G., Lee M.S. (2004). Effects of social support at work on depression and organizational productivity. Am. J. Health Behav..

[10] Kim K.W., Park S.J., Lim H.S., Cho H.H. (2017). Safety climate and occupational stress according to occupational accidents experience and employment type in shipbuilding industry of korea. Saf. Health Work.

[11] Zomer E., Rhee Y., Liew D., Ademi Z. (2021). The health and productivity burden of depression in south korea. Appl. Health Econ. Health Policy.

[12] Stress I.W. (2016). A Collective Challenge.

[13] Harandi T.F., Taghinasab M.M., Nayeri T.D. (2017). The correlation of social support with mental health: A meta-analysis. Electron. Physician.

[14] Weigl M., Stab N., Herms I., Angerer P., Hacker W., Glaser J. (2016). The associations of supervisor support and work overload with burnout and depression: A cross-sectional study in two nursing settings. J. Adv. Nurs..

[15] Hwang J.H., Choi S., Park H. (2015). Effects of job stress and supervisory support on depression of care givers in elderly care facilities. Korean J. Occup. Health Nurs..

[16] Park C.J., Yook J.H., Kim M.S., Lee D., Lim H.M., Hong Y.C. (2019). The association between quality of direct supervisor’s behavior and depressive mood in korean wage workers: The 4th korean working conditions survey. Ann. Occup. Environ. Med..

[17] Park J., Kim Y. (2020). Association of co-exposure to psychosocial factors with depression and anxiety in korean workers. J. Occup. Environ. Med..

[18] Hämmig O. (2017). Health and well-being at work: The key role of supervisor support. SSM Popul. Health.

[19] Baeriswyl S., Krause A., Elfering A., Berset M. (2017). How workload and coworker support relate to emotional exhaustion: The mediating role of sickness presenteeism. Int. J. Stress Manag..

[20] Age O. (2019). Working Better with Age.

[21] Wege N., Li J., Siegrist J. (2018). Are there gender differences in associations of effort-reward imbalance at work with self-reported doctor-diagnosed depression? Prospective evidence from the german socio-economic panel. Int. Arch. Occup. Environ. Health.

[22] Pudrovska T., Karraker A. (2014). Gender, job authority, and depression. J. Health Soc. Behav..

[23] Fan Z.J., Bonauto D.K., Foley M.P., Anderson N.J., Yragui N.L., Silverstein B.A. (2012). Occupation and the prevalence of current depression and frequent mental distress, wa brfss 2006 and 2008. Am. J. Ind. Med..

[24] Plaisier I., de Bruijn J.G., de Graaf R., ten Have M., Beekman A.T., Penninx B.W. (2007). The contribution of working conditions and social support to the onset of depressive and anxiety disorders among male and female employees. Soc. Sci. Med..

[25] Wang Y.P., Gorenstein C. (2015). Gender differences and disabilities of perceived depression in the workplace. J. Affect. Disord..

[26] Oliffe J.L., Phillips M.J. (2008). Men, depression and masculinities: A review and recommendations. J. Men Health.

[27] Gooding P.A., Hurst A., Johnson J., Tarrier N. (2012). Psychological resilience in young and older adults. Int. J. Geriatr. Psychiatry.

[28] Robb C., Haley W.E., Becker M.A., Polivka L.A., Chwa H.J. (2003). Attitudes towards mental health care in younger and older adults: Similarities and differences. Aging Ment. Health.

[29] Lips-Wiersma M., Wright S., Dik B. (2016). Meaningful work: Differences among blue-, pink-, and white-collar occupations. Career Dev. Int..

[30] Saraç M., Meydan B., Efil I. (2017). Does the relationship between person–organization fit and work attitudes differ for blue-collar and white-collar employees?. Manag. Res. Rev..

[31] Agarwal B., Brooks S.K., Greenberg N. (2020). The role of peer support in managing occupational stress: A qualitative study of the sustaining resilience at work intervention. Workplace Health Saf..

[32] Gilbreath B., Benson P.G. (2004). The contribution of supervisor behaviour to employee psychological well-being. Work Stress.

[33] Keus van de Poll M., Nybergh L., Lornudd C., Hagberg J., Bodin L., Kwak L., Jensen I., Lohela-Karlsson M., Torgén M., Bergstrom G. (2020). Preventing sickness absence among employees with common mental disorders or stress-related symptoms at work: A cluster randomised controlled trial of a problem-solving-based intervention conducted by the occupational health services. Occup. Environ. Med..

[34] Wu T.-Y., Hu C. (2009). Abusive supervision and employee emotional exhaustion: Dispositional antecedents and boundaries. Group Organ. Manag..

[35] Leung M.-Y., Liang Q., Olomolaiye P. (2016). Impact of job stressors and stress on the safety behavior and accidents of construction workers. J. Manag. Eng..

[36] Manuele F.A. (2011). Reviewing heinrich: Dislodging two myths from the practice of safety. Prof. Saf..

[37] Forastieri V. (2016). Prevention of psychosocial risks and work-related stress. Int. J. Labour. Res..

[38] Benavides F.G., Delclos J., Serra C. (2017). Welfare state and public health: The role of occupational health. Gac. Sanit..

[39] Kim Y.S., Rhee K.Y., Oh M.J., Park J. (2013). The validity and reliability of the second korean working conditions survey. Saf. Health Work.

