# Supplementary material for: Association between Self-Perceived Social Support in the Workplace and the Presence of Depressive/Anxiety Symptoms
Source: Int J Environ Res Public Health. 2021 Sep 30;18(19):10330. doi: 10.3390/ijerph181910330 (PMC8508368; doi:10.3390/ijerph181910330)
Supplement: Supplementary file 1 [file ijerph-18-10330-s001.zip › ijerph-1377421-supplementary.pdf]

Table S1. Multiple logistic regression model of depressive styptom and colleagues support.

Var	model 0	model 1	model 2	model 3
(Intercept)	0.02 (0.01-0.02)	0.01 (0.00-0.01)	0.01 (0.01-0.02)	0.01 (0.00-0.02)
**Support**				
Yes	1.00 (reference)	1.00 (reference)	1.00 (reference)	1.00 (reference)
No	1.65 (1.35-2.02)	1.65 (1.35-2.02)	1.61 (1.32-1.97)	1.61 (1.31-1.97)
**age**		1.02 (1.01-1.03)	1.02 (1.01-1.03)	1.02 (1.01-1.03)
**Sex**				
Male		1.00 (reference)	1.00 (reference)	1.00 (reference)
Female		1.11 (0.91-1.35)	1.00 (0.79-1.26)	1.01 (0.79-1.29)
**Income**				
Low			1.00 (reference)	1.00 (reference)
Low-Middle			0.74 (0.56-0.98)	0.72 (0.53-0.97)
High-Middle			0.67 (0.49-0.90)	0.66 (0.47-0.91)
High			0.74 (0.52-1.04)	0.73 (0.50-1.07)
**Education**				
Low			1.00 (reference)	1.00 (reference)
High			0.98 (0.78-1.23)	0.99 (0.77-1.28)
**Employment status**				
full-time				1.00 (reference)
part-time				1.15 (0.86-1.53)
**Work duration**				
<5year				1.00 (reference)
>=5year				1.03 (0.80-1.31)
**Working hour**				
<=40hours				1.00 (reference)
41-52hours				1.17 (0.93-1.47)
>=53hours				1.35 (0.99-1.84)
**Shift work**				
No				1.00 (reference)
Yes				1.01 (0.73-1.39)
**Occupational category**				
white-collar				1.00 (reference)
pink-collar				0.90 (0.68-1.19)
blue-collar				0.90 (0.66-1.22)

Table S2. Multiple logistic regression model of anxiety styptom and colleagues support

Var	model 0	model 1	model 2	model 3
(Intercept)	0.02 (0.02-0.03)	0.01 (0.01-0.02)	0.01 (0.01-0.02)	0.01 (0.01-0.02)
**Support**				
Yes	1.00 (reference)	1.00 (reference)	1.00 (reference)	1.00 (reference)
No	1.66 (1.39-1.98)	1.69 (1.41-2.01)	1.70 (1.42-2.03)	1.69 (1.41-2.02)
**age**		1.02 (1.01-1.03)	1.02 (1.01-1.03)	1.02 (1.01-1.03)
**Sex**				
Male		1.00 (reference)	1.00 (reference)	1.00 (reference)
Female		0.83 (0.70-0.99)	0.87 (0.71-1.07)	0.89 (0.71-1.10)
**Income**				
Low			1.00 (reference)	1.00 (reference)
Low-Middle			0.80 (0.61-1.04)	0.78 (0.59-1.04)
High-Middle			0.76 (0.58-1.01)	0.77 (0.57-1.04)
High			1.15 (0.85-1.55)	1.16 (0.83-1.62)
**Education**				
Low			1.00 (reference)	1.00 (reference)
High			0.92 (0.75-1.12)	0.99 (0.79-1.24)
**Employment status**				
full-time				1.00 (reference)
part-time				1.23 (0.94-1.59)
**Work duration**				
<5year				1.00 (reference)
>=5year				1.09 (0.87-1.35)
**Working hour**				
<=40hours				1.00 (reference)
41-52hours				1.47 (1.21-1.79)
>=53hours				1.32 (1.00-1.74)
**Shift work**				
No				1.00 (reference)
Yes				1.21 (0.93-1.58)
**Occupational category**				
white-collar				1.00 (reference)
pink-collar				1.04 (0.81-1.33)
blue-collar				0.95 (0.73-1.24)

Table S3. Multiple logistic regression model of depressive styptom and supervisor support

Var	model 0	model 1	model 2	model 3
(Intercept)	0.02 (0.01-0.02)	0.01 (0.00-0.01)	0.01 (0.01-0.02)	0.01 (0.00-0.02)
**Support**				
Yes	1.00 (reference)	1.00 (reference)	1.00 (reference)	1.00 (reference)
No	1.77 (1.45-2.16)	1.75 (1.44-2.14)	1.72 (1.40-2.10)	1.71 (1.40-2.09)
**age**		1.02 (1.01-1.03)	1.02 (1.01-1.03)	1.02 (1.01-1.03)
**Sex**				
Male		1.00 (reference)	1.00 (reference)	1.00 (reference)
Female		1.11 (0.91-1.35)	1.00 (0.79-1.27)	1.01 (0.79-1.29)
**Income**				
Low			1.00 (reference)	1.00 (reference)
Low-Middle			0.74 (0.56-0.98)	0.72 (0.54-0.98)
High-Middle			0.67 (0.49-0.91)	0.66 (0.47-0.91)
High			0.74 (0.53-1.05)	0.74 (0.50-1.08)
**Education**				
Low			1.00 (reference)	1.00 (reference)
High			0.99 (0.79-1.24)	1.00 (0.77-1.29)
**Employment status**				
full-time				1.00 (reference)
part-time				1.16 (0.87-1.55)
**Work duration**				
<5year				1.00 (reference)
>=5year				1.03 (0.80-1.31)
**Working hour**				
<=40hours				1.00 (reference)
41-52hours				1.17 (0.93-1.47)
>=53hours				1.34 (0.98-1.83)
**Shift work**				
No				1.00 (reference)
Yes				1.01 (0.73-1.38)
**Occupational category**				
white-collar				1.00 (reference)
pink-collar				0.91 (0.69-1.20)
blue-collar				0.90 (0.66-1.22)

Table S4. Multiple logistic regression model of anxiety styptom and supervisor support

Var	model 0	model 1	model 2	model 3
(Intercept)	0.02 (0.02-0.02)	0.01 (0.01-0.02)	0.01 (0.01-0.02)	0.01 (0.01-0.02)
**Support**				
Yes	1.00 (reference)	1.00 (reference)	1.00 (reference)	1.00 (reference)
No	1.89 (1.59-2.25)	1.90 (1.60-2.26)	1.92 (1.61-2.29)	1.91 (1.60-2.27)
**age**		1.02 (1.01-1.03)	1.01 (1.00-1.03)	1.01 (1.00-1.03)
**Sex**				
Male		1.00 (reference)	1.00 (reference)	1.00 (reference)
Female		0.83 (0.69-0.99)	0.88 (0.71-1.08)	0.89 (0.72-1.11)
**Income**				
Low			1.00 (reference)	1.00 (reference)
Low-Middle			0.80 (0.61-1.04)	0.79 (0.59-1.04)
High-Middle			0.77 (0.58-1.01)	0.77 (0.57-1.04)
High			1.16 (0.86-1.57)	1.18 (0.84-1.65)
**Education**				
Low			1.00 (reference)	1.00 (reference)
High			0.92 (0.76-1.13)	0.99 (0.79-1.25)
**Employment status**				
full-time				1.00 (reference)
part-time				1.23 (0.95-1.60)
**Work duration**				
<5year				1.00 (reference)
>=5year				1.09 (0.88-1.35)
**Working hour**				
<=40hours				1.00 (reference)
41-52hours				1.47 (1.20-1.78)
>=53hours				1.30 (0.99-1.72)
**Shift work**				
No				1.00 (reference)
Yes				1.21 (0.93-1.57)
**Occupational category**				
white-collar				1.00 (reference)
pink-collar				1.05 (0.82-1.34)
blue-collar				0.95 (0.73-1.23)
